# Time-to-Death Longitudinal Characterization of Clinical Variables and Longitudinal Prediction of Mortality in COVID-19 Patients: A Two-Center Study

**DOI:** 10.3389/fmed.2021.661940

**Published:** 2021-04-29

**Authors:** Anne Chen, Zirun Zhao, Wei Hou, Adam J. Singer, Haifang Li, Tim Q. Duong

**Affiliations:** ^1^Department of Radiology, Montefiore Health System and Albert Einstein College of Medicine, Bronx, NY, United States; ^2^Department of Radiology, Renaissance School of Medicine, Stony Brook University, Stony Brook, NY, United States; ^3^Department of Family Medicine, Renaissance School of Medicine, Stony Brook University, Stony Brook, NY, United States; ^4^Department of Emergency Medicine, Renaissance School of Medicine, Stony Brook University, Stony Brook, NY, United States

**Keywords:** prediction, SARS-CoV-2, longitudinal, trend, clinical variables

## Abstract

**Objectives:** To characterize the temporal characteristics of clinical variables with time lock to mortality and build a predictive model of mortality associated with COVID-19 using clinical variables.

**Design:** Retrospective cohort study of the temporal characteristics of clinical variables with time lock to mortality.

**Setting:** Stony Brook University Hospital (New York) and Tongji Hospital.

**Patients:** Patients with confirmed positive for severe acute respiratory syndrome coronavirus-2 using polymerase chain reaction testing. Patients from the Stony Brook University Hospital data were used for training (80%, *N* = 1,002) and testing (20%, *N* = 250), and 375 patients from the Tongji Hospital (Wuhan, China) data were used for testing.

**Intervention:** None.

**Measurements and Main Results:** Longitudinal clinical variables were analyzed as a function of days from outcome with time-lock-to-day of death (non-survivors) or discharge (survivors). A predictive model using the significant earliest predictors was constructed. Performance was evaluated using receiver operating characteristics area under the curve (AUC). The predictive model found lactate dehydrogenase, lymphocytes, procalcitonin, D-dimer, C-reactive protein, respiratory rate, and white-blood cells to be early predictors of mortality. The AUC for the zero to 9 days prior to outcome were: 0.99, 0.96, 0.94, 0.90, 0.82, 0.75, 0.73, 0.77, 0.79, and 0.73, respectively (Stony Brook Hospital), and 1.0, 0.86, 0.88, 0.96, 0.91, 0.62, 0.67, 0.50, 0.63, and 0.57, respectively (Tongji Hospital). In comparison, prediction performance using hospital admission data was poor (AUC = 0.59). Temporal fluctuations of most clinical variables, indicative of physiological and biochemical instability, were markedly higher in non-survivors compared to survivors (*p* < 0.001).

**Conclusion:** This study identified several clinical markers that demonstrated a temporal progression associated with mortality. These variables accurately predicted death within a few days prior to outcome, which provides objective indication that closer monitoring and interventions may be needed to prevent deterioration.

## Introduction

Coronavirus disease 2019 (COVID-19) has infected over 39 million people and killed more than 1 million people worldwide (October 18, 2020) (1–3). The widespread outbreaks with recent spikes across the states and the number of recurrences have strained and will continue to strain healthcare resources. There is an urgent need for effective tools for frontline physicians to effectively manage COVID-19 patients and anticipate resource needs under time-sensitive, stressful, and potentially resource-constrained circumstances in this pandemic.

Over a hundred commonly measured clinical variables associated with COVID-19 infection have been identified [see reviews (4–6)] including demographics, clinical signs and symptoms, comorbidities, serial imaging data, serial vital sign data, and serial laboratory blood tests, amongst others. A few studies have used some clinical variables at admission to the emergency department to predict likelihood of critical illness or mortality (7–17). However, patients presenting to hospitals are in various stages of disease severity. Prediction of mortality and other outcomes far downstream using only clinical variables at admission is likely inadequate. There is no consensus as to which clinical variables are the earliest predictors or most predictive of mortality (7–17). This is in part because patients presenting to hospitals are of variable disease severity and treatment of COVID-19 is still evolving. To our knowledge, there has been no systematic evaluation of the temporal characteristics of these clinical variables leading to mortality in COVID-19 and how these temporal characteristics are judiciously used to inform clinical decision making.

The goal of this study was thus to characterize the temporal progression of clinical and laboratory variables in COVID-19 patients with time lock to the day of death or discharge. We compared survivors and non-survivors to determine the earliest predictors of mortality in the disease progression. Based on these data, we then developed a mathematical model to predict mortality at each day prior to outcome using individual and combinations of these clinical predictors. This predictive model was developed and independently tested using data from Stony Brook University Hospital in New York. To extend its generalizability, we further tested this predictive model on an independent COVID-19 patient cohort from Tongji Hospital, Wuhan, China. To our knowledge, this is one of the first longitudinal models to monitor the progression and mortality in COVID-19.

## Materials and Methods

### Study Population

Our study followed the Transparent Reporting of a Multivariable Prediction Model for Individual Prognosis or Diagnosis (TRIPOD). Data came from two sites: The Stony Brook University Hospital (New York) data were used for training (80%) and testing (20%), while the Tongji Hospital (Wuhan, China) data were used for just testing purposes. This was a retrospective study approved by the Stony Brook University Institution Review Board Office of Research Compliance, approval number IRB2020-00207, and was exempt from informed consent and Health Insurance Portability & Accountability Act (HIPAA) waiver. The inclusion criteria were: (i) patients who were diagnosed by positive tests of real-time polymerase chain reaction (RT-PCR) for severe acute respiratory syndrome coronavirus 2 (SARS-CoV-2), and (ii) patients who were hospitalized. Exclusion criteria were: (i) COVID-19 patients who were under 18 years of age, and (ii) patients who were still in the hospital at the time of analysis. The COVID-19 Persons Under Investigation (PUI) registry from the Emergency Department consisted of 5,766 patients from February 7, 2020, and May 4, 2020. Of these patients, 2,594 were confirmed COVID-19 positive cases, of which 1252 were hospitalized. Primary analysis was performed on all hospitalized patients (*N* = 1,252, 14.5% mortality rate), and secondary analysis was performed on general floor (*N* = 1001, 8.09% mortality rate) and ICU (*N* = 251, 40.2% mortality rate) patient groups.

The Tongji Hospital (Wuhan, China) data were obtained from Jan 10, 2020 to Feb 24, 2020 (*N* = 485, of which 375 had the needed clinical variables) with approval of their institutional review board and waiver of informed consent (18). Of the 375 patients, 201 survived and 174 died (46.4% mortality rate). Similar inclusion and exclusion criteria were applied to these de-identified data. This dataset was used for “testing” only.

### Data Collection

The clinical outcome was mortality at discharge. The input variables included demographic information (age, gender, ethnicity, and race), chronic comorbidities (smoking, diabetes, hypertension, asthma, chronic obstructive pulmonary disease, coronary artery disease, heart failure, cancer, immunosuppression, and chronic kidney disease), serial vital signs (heart rate, respiratory rate, pulse oxygen saturation [SpO_2_], systolic blood pressure and temperature), and serial laboratory tests (C-reactive protein [CRP], D-dimer, ferritin, lactate dehydrogenase [LDH], lymphocytes, procalcitonin, alanine aminotransferase [ALT], brain natriuretic peptide [BNP], and troponin).

### Statistical Analysis and Predictive Modeling

Statistical analysis was performed using SPSS v26 (IBM, Armonk, NY) and SAS v9.4 (SAS Institute, Cary, NC). Group comparisons of categorical variables in frequencies and percentages were performed using the Chi-squared test or Fisher exact test. Group comparison of continuous variables in medians and interquartile ranges (IQR) used the Mann-Whitney *U* test. For all analyses, a *p* < 0.05 was considered to be statistically significant with correction for multiple comparisons with the false discovery rate where appropriate.

Clinical variables were analyzed as a function of days from outcome with time-locked to day of death (non-survivors) or discharge (survivors). Clinical variables were compared between groups at each time point with linear mixed models that included demographic information such as sex, age, ethnicity, and comorbidity as covariates. Within-subject correlation was adjusted in the linear mixed models using covariance (i.e., compound symmetric, autoregressive, or unstructured) matrices.

The temporal fluctuation of each clinical variable between groups was calculated by taking within-subject standard deviation across time normalized by mean, excluding the three time points closest to death or discharge to avoid possible spikes closer to the day of death. The medians of within-subject standard deviations were compared between the non-survivors and survivors using the Mann-Whitney *U* test.

Univariable logistic regression models were first built using individual clinical variables to predict outcomes on each day separately. Prediction performance was evaluated by area under the curve (AUC) of the receiver operating characteristic (ROC) curve. The Stony Brook University Hospital data were split into 80% for training and 20% for testing. The average ROC curve and AUC were obtained with five runs. Using ROC analysis, the top earliest predictors were identified. Instead of calibration measures (e.g., calibration slope), we demonstrated consistency through internal and external validation and systematically selected top variables for prediction. We started with univariable models (single predictor) and evaluated different combinations of 12 variables. We further constructed models using combinations of top predictors that included top three, top five, and top seven clinical variables. AUCs with the top 3, 5, or 7 variables for all three analysis cohorts were analyzed to verify consistency of top predictors across models. These were done for different days prior to outcome separately. For comparison, prediction performance using clinical variables at admission of the same dataset was also computed. In addition, Tongji Hospital data were also used as a “testing” dataset for external validation.

To avoid the potential of overfitting, for training, we performed univariable analysis first and identified 12 laboratory measures and vital signs to build the predictive model to predict mortality. Then we performed variable selection and used top 3, 5, or 7 variables to present results. No more than 10 variables were included in one model at any given time for a cohort of 1,252 patients.

## Results

### Clinical Variables

Of the 1,252 hospitalized patients (Stony Brook Hospital), 1,070 survived and 182 did not (14.5% mortality rate). Table 1 summarizes the demographics, comorbidities, vital signs, and laboratory data of the survivors and non-survivors. The non-survivor group was older than the survivor group (73 ± 15 vs. 60 ± 17 years of age, *p* < 0.0001), with more males dying than females (*p* = 0.021). Ethnicity and race were statistically different between groups (*p* < 0.05). History of smoking, hypertension, chronic obstructive pulmonary disease (COPD), coronary artery disease, and heart failure were significantly different between groups (*p* < 0.05, after correction for multiple comparisons). Signs and symptoms such as fever, cough, fatigue, myalgia, nausea or vomiting, and chest discomfort were significantly different between groups (*p* < 0.05, after correction for multiple comparisons). The non-surviving group had reported greater co-morbidities, it was surprising to find that the surviving cohort reported more signs and symptoms.

**Table 1:** All hospitalized patients.

	Non-survivors (*N =* 182)	Survivors (*N =* 1070)	***p* value**
**Demographics**			
Age	73.08 ± 14.56	59.87 ± 16.94	<0.0001^***^
Sex			0.021^*^
Female	65 (35.7%)	480 (44.9%)	
Male	117 (64.3%)	590 (55.1%)	
Ethnicity			0.002^**^
Hispanic/Latino	29 (15.9%)	290 (27.1%)	
Non-hispanic/Latino	131 (72.0%)	632 (59.1%)	
Unknown	22 (12.1%)	148 (13.8%)	
Race			0.033^*^
Caucasian	114 (62.6%)	561 (52.4%)	
African-American	9 (5.0%)	81 (7.6%)	
Others	59 (32.4%)	428 (40.0%)	
**Comorbidities**^**a**^			
Smoking	69 (37.9%)	260 (24.3%)	0.0003^*^
Diabetes	60 (33.1%)	277 (25.9%)	0.060
Hypertension	120 (66.3%)	494 (46.3%)	<0.0001^*^
Asthma	8 (4.4%)	62 (5.8%)	0.500
COPD	28 (15.5%)	83 (7.8%)	0.002^*^
Coronary artery disease	56 (30.9%)	133 (12.5%)	<0.0001^*^
Heart failure	37 (20.4%)	64 (6.0%)	<0.0001^*^
Cancer	23 (12.7%)	96 (9.0%)	0.140
Immunosuppression	13 (7.2%)	83 (7.8%)	0.780
Chronic kidney disease	25 (13.8%)	93 (8.7%)	0.050
**Signs and symptoms**^**a**^			
Fever	99 (54.4%)	697 (65.1%)	0.020^*^
Cough	90 (49.5%)	720 (67.3%)	<0.0001^*^
Shortness of breath	126 (69.2%)	688 (64.3%)	0.280
Fatigue	27 (14.8%)	251 (23.5%)	0.020^*^
Sputum	16 (8.8%)	70 (6.5%)	0.340
Myalgia	18 (9.9%)	250 (23.4%)	0.0002^*^
Diarrhea	32 (17.6%)	243 (22.7%)	0.190
Nausea or vomiting	12 (6.6%)	231 (21.6%)	<0.0001^*^
Sore throat	7 (3.8%)	76 (7.1%)	0.180
Rhinorrhea	6 (3.3%)	48 (4.5%)	0.540
Loss of smell	7 (3.8%)	41 (3.8%)	0.990
Loss of taste	7 (3.8%)	52 (4.9%)	0.590
Headache	9 (4.9%)	99 (9.3%)	0.110
Chest discomfort	12 (6.6%)	188 (17.6%)	0.0007^*^

*Demographics, comorbidities and symptoms of COVID-19 patients who did and did not survive*.

a *P values were adjusted with the False Discovery Rate*.

* *p < 0.05*,

** *p < 0.01*,

*** *p < 0.001*.

**Figure 1:**
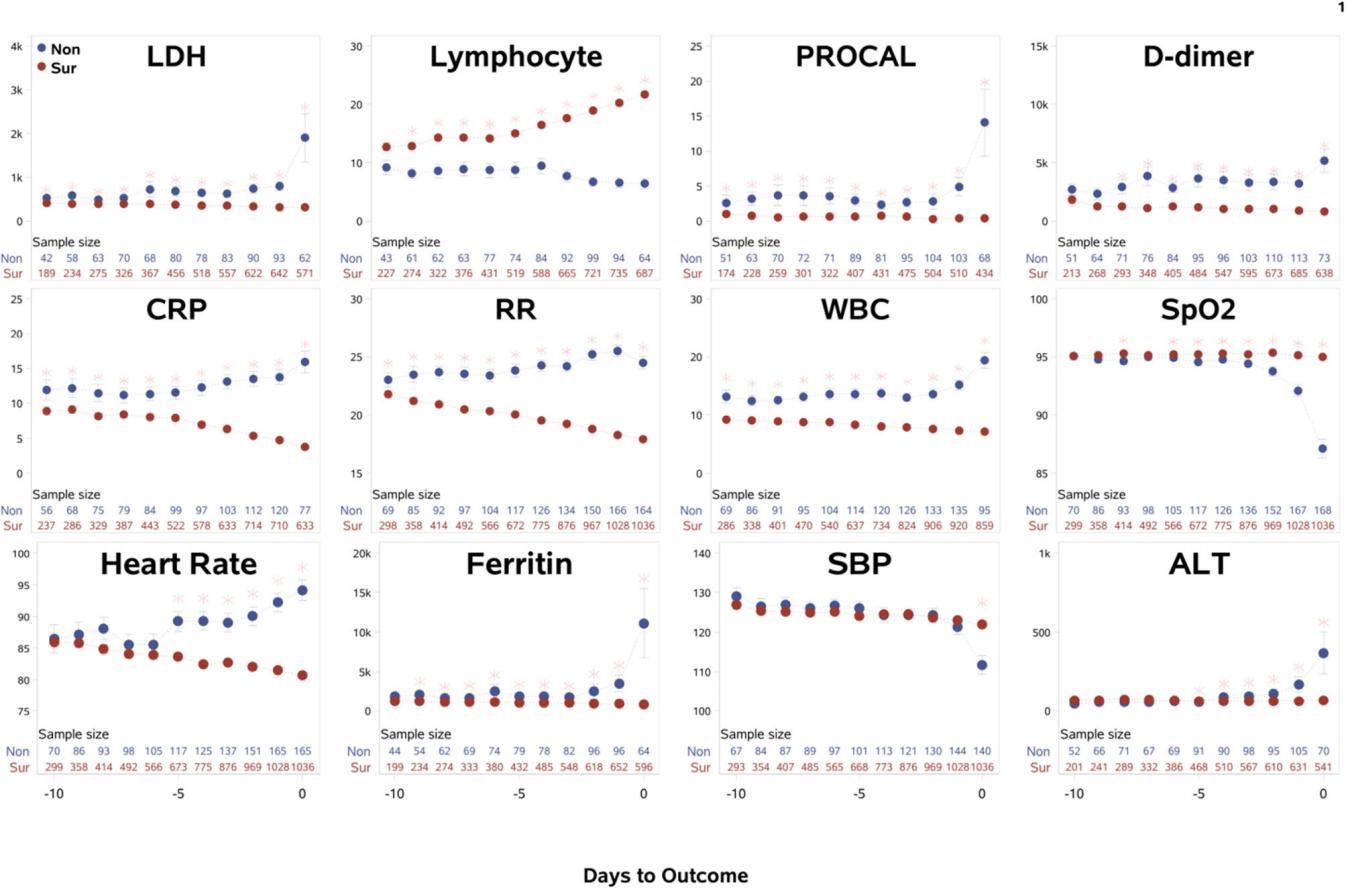
The time courses of the clinical variables of all hospitalized patients as a function of days to outcome, time lock to the day of death (“Non”: non-survivors) or the day of discharge (“Sur”: survivors). Error bars are SEM. Two rows of numbers are sample sizes. * indicates significant difference with correction of multiple comparison and covariate with sex, age, ethnicity, and comorbidities. BNP and troponin were not analyzed because their sample sizes were small and highly scattered.

The time course of the clinical variables as a function of days to outcome are shown in Figure 1. LDH, procalcitonin, ferritin, ALT, and SpO_2_ of non-survivors changed sharply on the day of or a day prior to death, relative to those of survivors. By contrast, lymphocyte count, CRP, respiratory rate, WBCs, and heart rate showed gradually increasing differences early on prior to death. Unexpectedly though, in the surviving group, ferritin and procalcitonin remained relatively elevated but stable throughout the entire hospitalization until discharge, despite being acute phase reactants. In addition, when looking at CRP and respiratory rate, it was noted that in the surviving cohort the values continued to decrease toward normal values rather than stay elevated like in the mortality cohort. This kind of temporal progression change was not evident in other temporal variables. Overall, there were more temporal fluctuations in the group that passed away as compared to the group that survived. We also separated the temporal characteristics of clinical variables of patients in the ICU cohort (*N* = 251, 40.2% mortality rate) and the general floor cohort (*N* = 1,001, 8.09% mortality rate) (Supplementary Figure 1). While there were some differences, the majority of the temporal characteristics leading up to mortality or discharge were overall similar amongst the ICU cohort, general floor cohort and all hospitalized patients, except that LDH, ferritin, and ALT spiked in the ICU cohort but not in the general floor cohort. As was supported by the trend of variables for all hospitalized patients, lymphopenia continued to be worse in the non-surviving cohort in both the ICU and the general floor. CRP and respiratory rate also continued to down-trend throughout hospital stay in the surviving cohort in the ICU and general floor.

The within-subject standard deviations of the clinical variables across time were computed to evaluate temporal fluctuation. For all hospitalized patients, the ratio of temporal fluctuation of non-survivors to survivors for D-dimer, procalcitonin, ferritin, WBCs, LDH, respiratory rate, CRP, SpO_2_, heart rate, and systolic blood pressure were 4.78, 3.75, 2.26, 2.15, 1.77, 1.63, 1.58, 1.55, 1.43, and 1.37, respectively (*p* < 0.001), indicating higher physiological instability amongst non-survivors compared to survivors. Similar results were found when data were separated into general floor and ICU patient groups.

**Figure 2:**
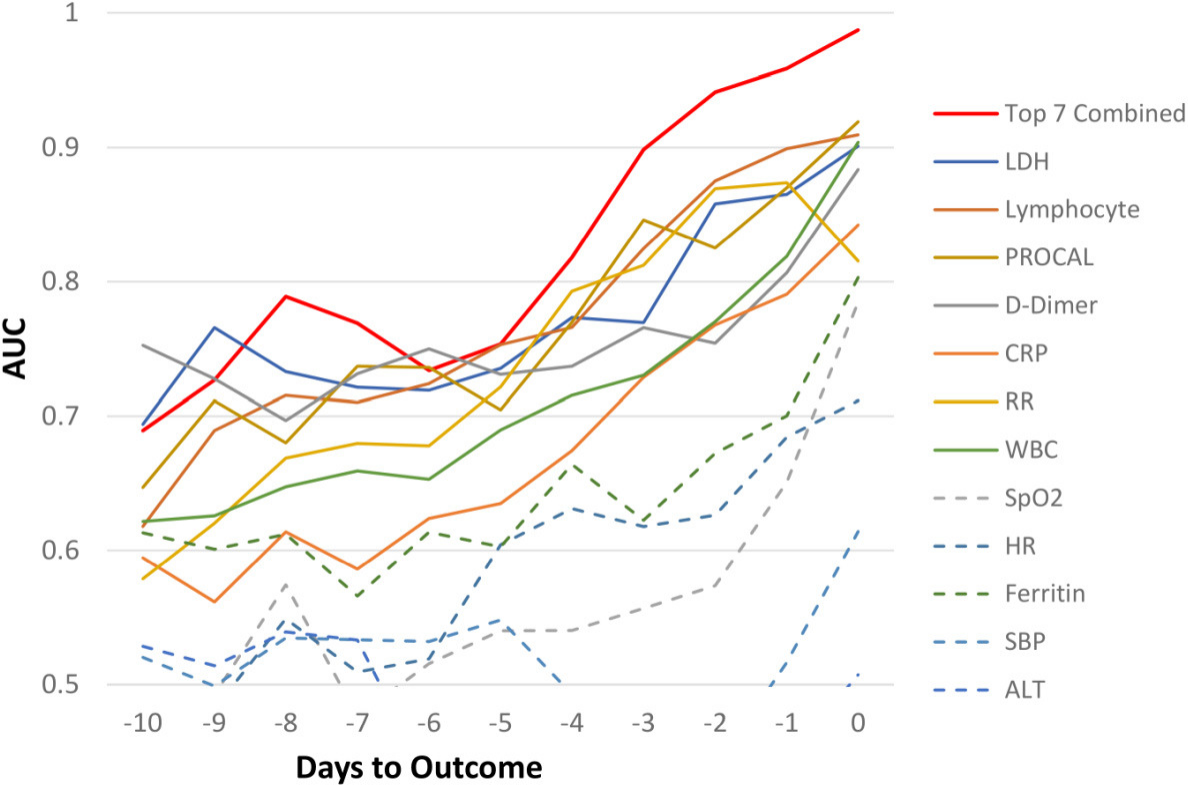
AUC for all hospitalized COVID-19 patients as a function of days to outcome, time lock to the day of death (non-survivors) or the day of discharge (survivors) for individual and top earliest predictors of mortality (“testing” data).

For the Stony Brook Hospital data, the AUC predicting mortality in all hospitalized patient cohort for individual clinical variables at each day are illustrated in Figure 2. Overall, we noticed the trend that the prediction performance, as determined by a higher AUC value, increased the closer we were to the outcome of death, with an AUC of 80–99% in days 0–4 prior to death, and AUC >70% from days 5–10 prior to death, with specificity higher than sensitivity. The earliest predictors that showed high prediction performance by AUC were LDH, lymphocytes, procalcitonin, D-dimer, CRP, respiratory rate, and WBCs. The AUC of combined top 7 predictors from zero to 9 days prior to outcome were: 0.99, 0.96, 0.94, 0.90, 0.82, 0.75, 0.73, 0.77, 0.79, and 0.73, respectively. The corresponding specificity were >0.96 for all zero to 9 days prior to outcome. The corresponding sensitivity was 0.80, 0.73, 0.68, 0.46, 0.34, 0.23, 0.12, 0.37, 0.33, and 0.29 respectively. The moderate to low sensitivity was due to data asymmetry, namely, low mortality rate (14.5%, all hospitalized COVID-19 patients), as expected. The sensitivity of ICU group with 40% mortality was excellent. With more multi-institutional datasets, the model should yield better sensitivity and generalizability.

**Figure 3:**
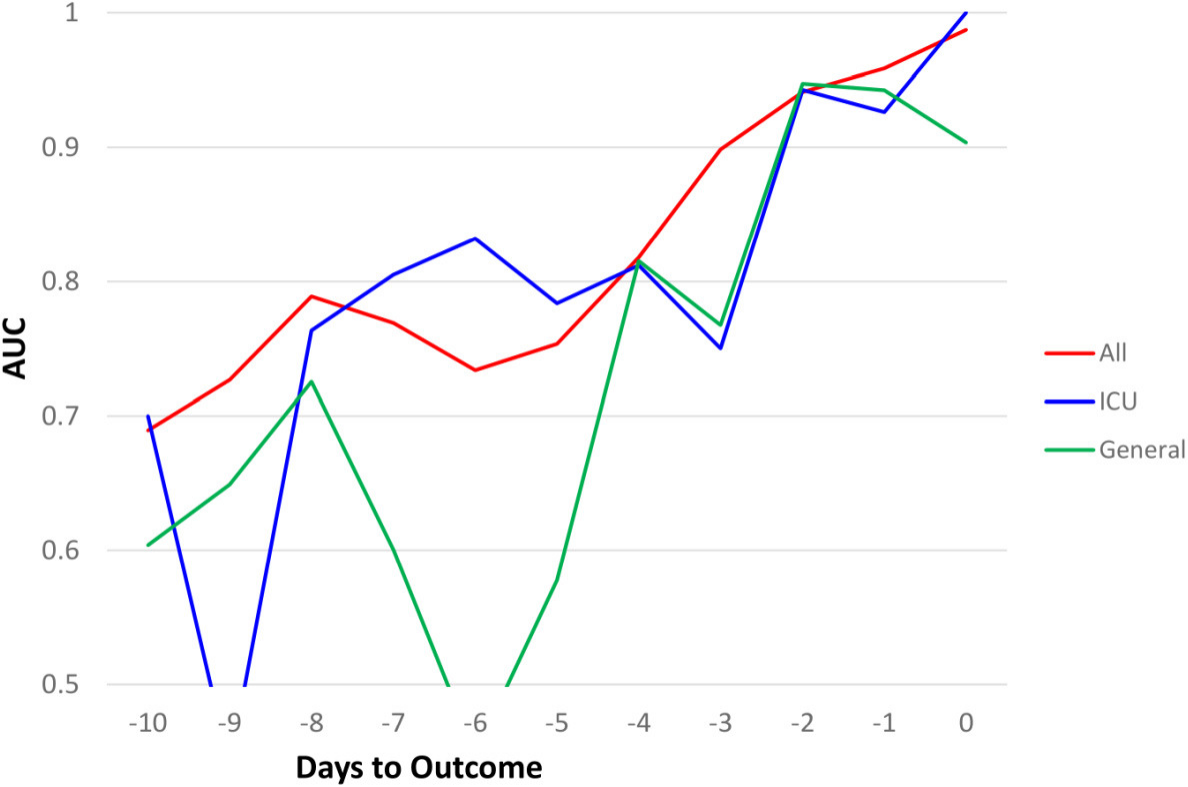
AUC comparisons of all hospitalized (*N* = 1252), ICU (*N* = 251), and general floor (*N* = 1,001) as a function of days to outcome, time lock to the day of death (non-survivors) or the day of discharge (survivors) for the seven earliest predictors of mortality (20% “testing” data).

Similar predictive models were built for the general floor cohort and the ICU cohort (Figure 3). The AUCs of top 7 predictors for all hospitalized cohort were better than that of ICU group, which in turn were better than general floor cohort. Prediction performance was consistent for the ICU group because data were more balanced (mortality rate of 40.2%) compared to the general floor group (mortality rate of 8.09%).

To determine the best predictive model, we built and tested predictive models with different combination of top clinical variables. AUC for predicting mortality from top 3 clinical variables (LDH, lymphocytes, and procalcitonin), top 5 clinical variables (LDH, lymphocytes, procalcitonin, D-dimer, and CRP) and top 7 clinical variables (LDH, lymphocytes, procalcitonin, D-dimer, CRP, respiratory rate, and WBCs) are listed in Table 2.

**Table 2:** Predictive performance of top 3, 5, and 7 clinical variables for all hospitalization, general floor, and ICU cohorts.

	**All (*****N=*** **1,252)**			**General Floor (*****N=*** **1,001)**			**ICU (*****N=*** **251)**		
**Day**	**AUC Top 3**	**AUC Top 5**	**AUC Top 7**	**AUC Top 3**	**AUC Top 5**	**AUC Top 7**	**AUC Top 3**	**AUC Top 5**	**AUC Top 7**
0	0.88	0.90	0.99	0.80	0.84	0.90	0.91	0.92	1.00
−1	0.79	0.81	0.96	0.70	0.74	0.94	0.89	0.85	0.93
−2	0.77	0.76	0.94	0.71	0.74	0.95	0.84	0.81	0.94
−3	0.66	0.67	0.90	0.58	0.53	0.77	0.87	0.72	0.75
−4	0.53	0.56	0.82	0.50	0.48	0.82	0.71	0.58	0.81
−5	0.56	0.61	0.75	0.57	0.53	0.58	0.61	0.69	0.78
−6	0.57	0.58	0.73	0.52	0.53	0.45	0.72	0.74	0.83
−7	0.59	0.59	0.77	0.50	0.50	0.60	0.77	0.69	0.81
−8	0.60	0.64	0.79	0.50	0.50	0.73	0.66	0.76	0.76
−9	0.59	0.58	0.73	0.52	0.52	0.65	0.59	0.56	0.44
−10	0.52	0.57	0.69	0.49	0.56	0.60	0.61	0.74	0.70

*The top 3-variable model includes LDH, lymphocytes, and procalcitonin, the top 5 variable model includes LDH, lymphocytes, procalcitonin, D-dimer, and CRP. The top 7 variable model includes LDH, lymphocytes, procalcitonin, D-dimer, CRP, respiratory rate, and WBCs*.

These models were designed by a statistical logistic regression model utilizing ROC analysis to identify which of the clinical variables or combination of clinical variables were the most predictive. It is not a scoring system. As a result, having missing variables like procalcitonin would likely reduce the prediction performance, but does not invalidate the use of these models. Prediction using the top 7 variables performed better than models using the top 5, which performed better than models using the top 3 variables. As a result, if a hospital is not able to collect certain lab values on patients, they could use the top 3 or top 5 variable prediction models, with the knowledge that it would not be as accurate if all seven variables were collected. This model requires further validation using large and multi-institutional dataset to achieve generalizability. With more data, the model should become more accurate.

By comparison, the prediction performance of the top individual clinical variables at admission to the emergency department yielded an AUC ranging from 0.50 to 0.61, and the combined earliest predictors at admission yielded an AUC of 0.59.

**Figure 4:**
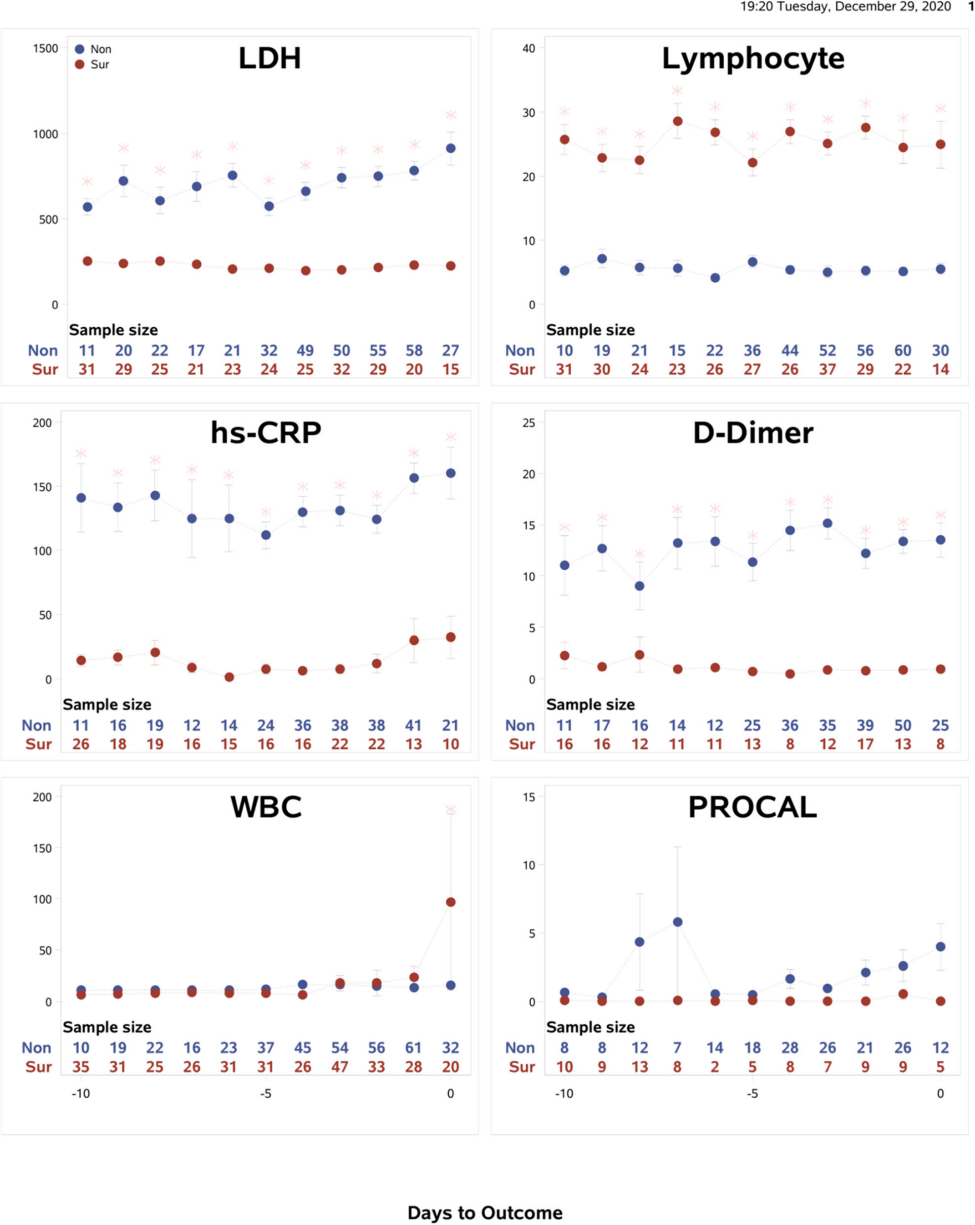
Tongji data: The time courses of the clinical variables as a function of days to outcome, time lock to the day of death (“Non”: non-survivors) or the day of discharge (“Sur”: survivors). Error bars are SEM (*N* = 375). Two rows of numbers are sample sizes. * indicates significant difference with correction of multiple comparison and covariate with sex and age.

Temporal characteristics of clinical variables were also described and compared between non-survivors and survivors for data from the Tongji Hospital, Wuhan, China (*N* = 375) (Figure 4). The mortality rate of this cohort was 46.4%. Some temporal characteristics were similar, and others were different from the Stony Brook data. In particular, LDH, lymphocytes, CRP and D-dimer showed significant differences between non-survivors and survivors from the onset and these differences were time-invariant. For external validation, a logistic regression model trained on the Stony Brook hospital data and tested on the Tongji Hospital data using the significant variables LDH, lymphocytes, CRP and D-dimer demonstrated the AUC for the zero to 9 days prior to death as follows: 1.0, 0.86, 0.88, 0.96, 0.91, 0.62, 0.67, 0.50, 0.63, and 0.57, respectively.

## Discussion

With widespread COVID-19 outbreaks, improved understanding of the temporal disease progression can guide prognosis and treatment, as well as anticipate resource needs. This study characterized the temporal progression of clinical variables in COVID-19 patients with time lock to the day of death or discharge. The major findings were: (i) the earliest predictors of mortality were lactate dehydrogenase, lymphocyte count, procalcitonin, D-dimer, C-reactive protein, respiratory rate, and white-blood cells, (ii) there is a down-trending CRP (normal limit <10 mg/L) and respiratory rate (normal limit 12–16 breaths/minute) to normal values in the survival cohort that is not evident in other temporal variables, (iii) the temporal fluctuations of most clinical variables, indicative of physiological and biochemical instability, were markedly higher in non-survivors compared to survivors, (iv) the overall performance of predictive models was better in the days leading up to the day of death, (v) the best predictive models were those using the top 7 variables, followed by the top 5 variables, and then by the top 3 variables, (vi) these predictive models were further tested on data from another hospital, and showed similar performance accuracy, and (vii) by comparison, prediction performance of the top individual clinical variables at hospital admission was poor.

While there are many COVID-19 prediction models (7–17), this study is novel because: (i) our predictive model assesses predictions at multiple time points with time locked to death, and (ii) it evaluated models using all hospitalized, general floor and ICU patients as well as data from another hospital to improve generalizability. This is in contrast to most previous COVID-19 prediction models that lacked external validation and used only admission data (one time point) which is less informative because patients come to hospitals at different degrees of severity. To our knowledge, this is the first study that systematically characterizes the longitudinal progression of commonly measured clinical variables over the course of hospitalization of COVID-19 patients.

### Temporal Progression of Clinical Variables

We characterized commonly measured clinical parameters into three categories: the variables that changed early on during the hospital course then gradually worsened (CRP, lymphocyte count, WBCs, respiratory rate, and heart rate), the variables that spiked before death (LDH, procalcitonin, ferritin, alanine aminotransferase, and SpO_2_ levels), and others that were time invariant.

CRP and lymphocyte count were found to be early warning signs of COVID-19 mortality. CRP, an acute inflammatory marker, was significantly elevated early in the hospitalization in the non-survivor group than the survivor group. In addition, patients who survived had declining levels of CRP throughout the hospital stay. These findings are consistent with previous reports on the predictive value of CRP in hospitalized COVID-19 patients (19, 20). However, our results suggest that the dynamic trend of CRP over time, rather than a single value, is predictive of outcome. One other study also correlated upward trending CRP with the eventual need of intubation and suggests that early rise in CRP predicts worse prognosis (21). As trending CRP is a widely accessible clinical tool, early CRP trends can assist physicians to stratify patients and determine the need for further medical intervention vs. symptomatic management alone.

Lymphopenia was also found to be an early predictor of mortality, consistent with literature review (22, 23). We found that in non-survivors, lymphocyte count is lower early in admission, and continues to trend downwards during the hospitalization. As lymphocytes play a significant role in the immune defense to viral infection, lymphopenia may reveal disease mechanisms of COVID-19 and suggest therapeutic targets. Presumed theories of lymphopenia include: (1) the virus directly damaging lymphocytes through coronavirus receptors, or lymphatic organs such as the lymph nodes and the spleen; (2) lymphocyte apoptosis induced by either inflammatory cytokine, metabolic derangements, or both (22, 24). Persistent lymphopenia causing mortality from COVID-19 infection could be a result of any one of these possible mechanisms and deserves further research. Nevertheless, our results confirmed the clinical utility of trending CRP and lymphocyte count in monitoring COVID-19 severity and risk stratification.

In contrast to the early indicators, LDH, procalcitonin, ferritin, alanine aminotransferase spiked by a few orders of magnitude prior to death but largely remained temporally stable and elevated during hospitalization. Elevation of these markers indicate significant oxidative stress and systemic inflammation, particularly prior to death. LDH, in particular, has been found to be associated with mortality in respiratory epidemics of MERS-CoV, H7N9, and H5N1 (25). Together with procalcitonin and ferritin, the spikes of these physiological parameters demonstrate that the dynamic inflammatory response elicited by COVID-19 has a key role affecting disease severity and outcome. Ferritin and procalcitonin are acute phase reactants, so it is logical that they would spike with the deterioration of patients. Unexpectedly, in the surviving group, ferritin and procalcitonin remained relatively elevated but stable throughout the entire hospitalization until discharge, despite being acute phase reactants. This suggests that patients with COVID-19 remain at a hyper-inflammatory state even upon discharge, which has implications for post-discharge follow-up and treatments. Multiple studies have also demonstrated elevation of additional inflammatory markers such as IL-6, IL-8, IL-10, and TNF-α in COVID-19 patients (26, 27). Our results suggest that severe COVID-19 infection is more inflammatory than milder disease, and that mortality from COVID-19 is associated with an overwhelming inflammatory response.

### Variables Predictive of Outcome

Understanding the temporal progression of these clinical markers allowed us to construct a prediction model with remarkable performance. Our model identified the earliest predictors of death to be LDH, lymphocytes, procalcitonin, D-dimer, CRP, respiratory rate, and WBCs. The predictive model using these combined predictors yielded a remarkable prediction performance: 80–99% AUC 0 to 4 days prior to death, and >70% AUC from 5 to 10 days prior to death, with specificity higher than sensitivity.

Compared to existing prediction models of COVID-19, our model has several strengths. This is the first time that temporal progression of clinical variables is considered into a prediction model. Most published models used clinical data at admission (7–9, 9–17, 28). However, we found that prediction using the admission timepoint has relatively poor accuracy compared to a few days prior to outcome. While this finding is intuitively logical, we provided evidence that roughly 4 days prior to outcome, our current model can yield a highly accurate prediction. Therefore, our prediction model may aid clinicians to anticipate patient's care escalation with a concrete timeline. From the top 7 predictors, we also derived prediction using the top 3, and top 5 predictors. Depending on the availability of laboratory tests, the number of input clinical variables can be customized at different resource settings for wider applicability.

We also sought external validation on a dataset from Tongji hospital in Wuhan, China. Some temporal characteristics were similar, and others were different from the Stony Brook data. LDH, lymphocytes, CRP and D-dimer showed differences between non-survivors and survivors from the onset and these differences were time-invariant, which may be indicative of a more severely ill cohort. This is also consistent with a higher mortality rate of 46.4% in Tongji cohort, compared to 14.5% in the Stony Brook cohort. Nevertheless, using our predictive model, the AUCs for the zero to 9 days prior to death were better than AUCs of Stony Brook data. This may be attributive to the high mortality rate. Despite the different populations of COVID-19 patients, external validation of our model supports the notion that these top earliest predictors of mortality are likely generalizable.

### Clinical Implications

While the focus of our paper was to analyze the temporal progression of clinical variables to find which ones were predictive of mortality, we believe our findings have clinical relevance. Our prediction model can assist physicians to make decisions based on common laboratory values in as early as 10 days prior to death. As a result, these variables may serve as an early warning of the poor prognosis later on and the need to intervene now, whether by initiating dexamethasone treatment or starting prone positioning, if not already done so, as early intervention has been associated with lower mortality (29, 30). Currently, as there is no curative drug yet, symptomatic treatment through Supplementary oxygen or anticoagulation has been the mainstay methodology of treatment in hospitals. With the knowledge that COVID-19 induces a hyper-coagulable state, anticoagulation (i.e., low molecular weight heparin) has become an important treatment in the acute and long-term setting (31). However, because the use of anticoagulation comes with side effects such as bleeding, the decision of when to initiate anticoagulation has been contentious (31). With our predictive model, physicians may objectively weigh the risk and benefit of initiating anticoagulation with the predicted outcome. In addition, our findings suggests that the trend of key clinical variables such as CRP and lymphocytes can be used as treatment response to monitor treatment progress.

While our model and other similar models to date are not yet be able to predict mortality of an individual patient at this time, it is nonetheless important to objectively determine which set of variables are most predictive of outcomes. These variables were determined from group data analysis instead of depending on the variable experience of each individual physician. With further testing and validation as more standardized COVID-19 datasets or predictive models are shared publicly, predicting mortality and other outcomes on an individual patient may be possible.

Our study has several novelties: This is the first study that systematically characteristic the temporal progression of clinical variables with time-lock to the day of death or discharge. Most previous studies (7–17) reported similar laboratory variables to be prediction of mortality but based on only laboratory variables at hospital admission, which we believe to be less accurate because they were far downstream.

Using these temporal characteristics, we determined that the earliest predictors of mortality were lactate dehydrogenase, lymphocyte count, procalcitonin, D-dimer, C-reactive protein, respiratory rate, and white-blood cells, and that they could accurately predicted a few days prior to death. Another novelty is that we have designed prediction models using the top 7 variables, the top 5 variables, and the top 3 variables. We found that accuracy, as determined by a higher AUC value, was highest when using the top 7 variables, and lowest when using just the top 3 variables to predict outcome. Another interesting finding is that the high temporal fluctuations of many clinical variables, indicative of physiological and biochemical instability, were associated with higher likelihood of mortality. These findings were replicated on data from another hospital.

## Limitations

Our study has several limitations. A major limitation of the current study is its retrospective nature which could have unintentional patient selection bias. This study design is also subject to residual confounding factors that were unaccounted for. Our predictive model is based on data from two medical centers and requires further, multi-center prospective validation. We did not compare clinical variables to normal ranges in order to focus the analysis on differences between survivors and non-survivors. Our use of the logistic regression model instead of a proportional hazard model did not allow for evaluating survival time and comparison of variables across time points. In a future model, we will look at multiple time points across time and survival time. Radiological imaging data are not included (32–37). AUROC is not actionable for clinicians. We believe that given the large complexity and multiple clinical parameters, there is unlikely to be a single (or two) variable that can provide actionable insights for the bedside clinician. A model of collection of variables will likely be needed to predict mortality and we will provide a user-friendly Excel to provide insights that are potentially actionable. This model may have more utility in the general floor patients than ICU patients because unlikely ICU patients who are more closely monitored so their physiologic derangements are more evident rapidly, general floor patients are not monitored as closely. On the general floor, our predictive model could provide early warning signs for escalate care, and our knowledge of the temporal change in variables could be used to trend efficacy of treatment.

## Conclusion

This study characterized the temporal progression of readily available clinical and laboratory variables associated with COVID-19 infection, providing important insights in disease pathogenesis. The earliest clinical predictors of mortality were identified, and they accurately predicted death a few days prior to outcome. The indicators that change early on and gradually worsen can serve as early warning signs because they allow physicians to intervene and, thus, should be closely monitored in COVID-19 patients. This approach may prove useful for management of COVID-19 patients and allocation of hospital resources in time-sensitive, stressful, and resource-constrained circumstances.

## Data Availability Statement

De-identified dataset available to be shared upon request.

## Ethics Statement

The studies involving human participants were reviewed and approved by Stony Brook University Institution Review Board Office of Research Compliance. Written informed consent for participation was not required for this study in accordance with the national legislation and the institutional requirements.

## Author Contributions

AC, ZZ, and WH contributed equally to the collection of the data, analysis of the data, and the writing of this manuscript. AS, HL, and TD contributed equally to the collection and interpretation of the data, and in writing the manuscript. All authors contributed to the article and approved the submitted version.

## Conflict of Interest

The authors declare that the research was conducted in the absence of any commercial or financial relationships that could be construed as a potential conflict of interest.

## Supplementary Material

Supplementary Figure 1 The time courses of the clinical variables for **(A)** ICU group and **(B)** general floor group as a function of days to outcome, time lock to the day of death (“Non”: non-survivors) or the day of discharge (“Sur”: survivors). Error bars are SEM. Two rows of numbers are sample sizes. * indicates significant difference with correction of multiple comparison and covariate with sex, age, ethnicity and comorbidities. Patients upgraded to ICU from a general floor were included in the ICU group.
