# Supplementary figures and images for: Time-to-Death Longitudinal Characterization of Clinical Variables and Longitudinal Prediction of Mortality in COVID-19 Patients: A Two-Center Study
Source: Front Med (Lausanne). 2021 Apr 29;8:661940. doi: 10.3389/fmed.2021.661940 (PMC8116568; doi:10.3389/fmed.2021.661940)

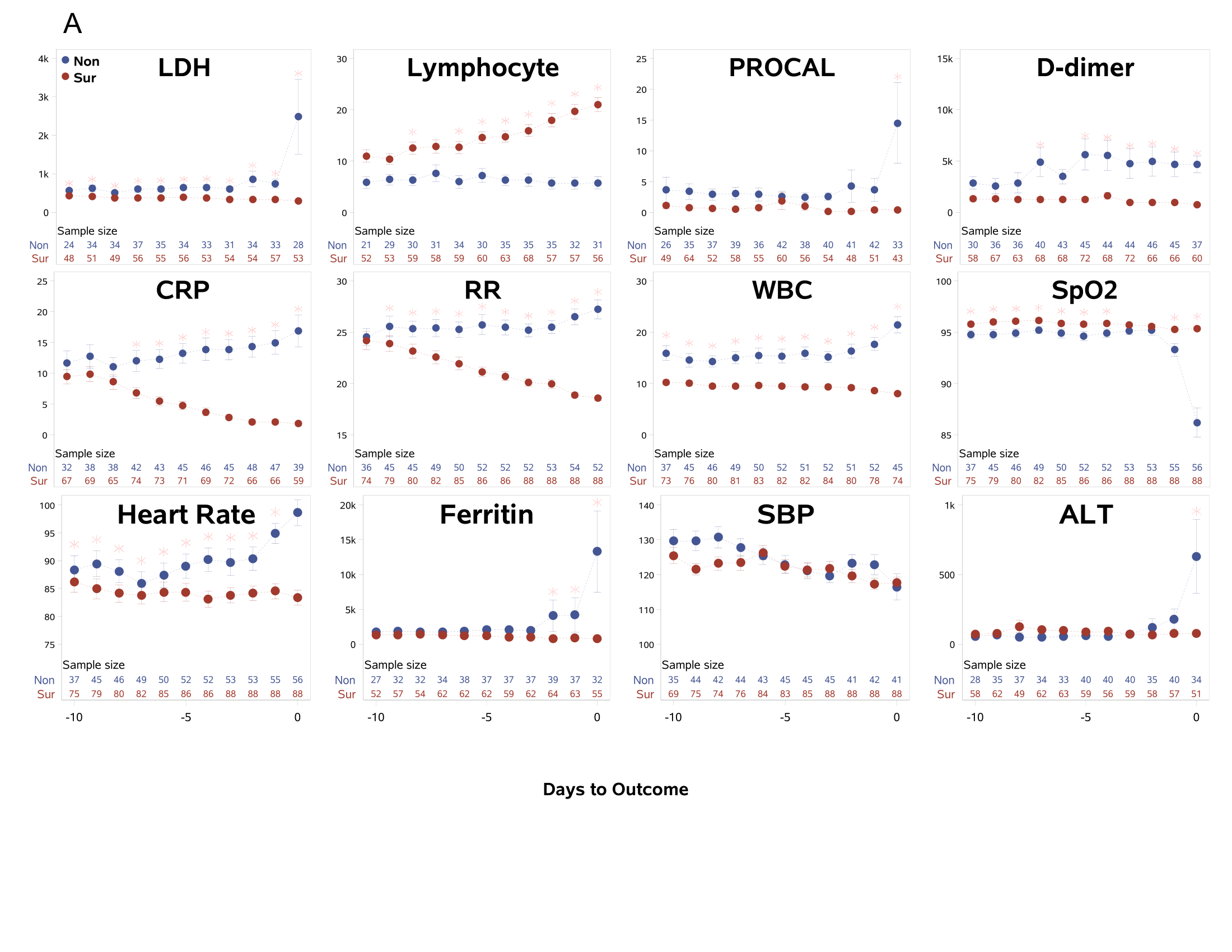

Supplement: Supplementary Figure 1 — The time courses of the clinical variables for (A) ICU group and (B) general floor group as a function of days to outcome, time lock to the day of death (“Non”: non-survivors) or the day of discharge (“Sur”: survivors). Error bars are SEM. Two rows of numbers are sample sizes. * indicates significant difference with correction of multiple comparison and covariate with sex, age, ethnicity and comorbidities. Patients upgraded to ICU from a general floor were included in the ICU group. [file Image_1.TIFF]
